# Single-Pixel Imaging and Its Application in Three-Dimensional Reconstruction: A Brief Review

**DOI:** 10.3390/s19030732

**Published:** 2019-02-11

**Authors:** Ming-Jie Sun, Jia-Min Zhang

**Affiliations:** School of Instrumentation Science and Optoelectronic Engineering, Beihang University, Beijing 100191, China; jiamin960505@163.com

**Keywords:** single-pixel imaging, ghost imaging, compressive sensing, three-dimensional imaging, time-of-flight, depth mapping, stereo vision

## Abstract

Whereas modern digital cameras use a pixelated detector array to capture images, single-pixel imaging reconstructs images by sampling a scene with a series of masks and associating the knowledge of these masks with the corresponding intensity measured with a single-pixel detector. Though not performing as well as digital cameras in conventional visible imaging, single-pixel imaging has been demonstrated to be advantageous in unconventional applications, such as multi-wavelength imaging, terahertz imaging, X-ray imaging, and three-dimensional imaging. The developments and working principles of single-pixel imaging are reviewed, a mathematical interpretation is given, and the key elements are analyzed. The research works of three-dimensional single-pixel imaging and their potential applications are further reviewed and discussed.

## 1. Introduction

Image retrieval has been an important research topic since the invention of cameras. In modern times, images are usually retrieved by forming an image with a camera lens and recording the image using a detector array. With the rapid development of complementary metal-oxide-semiconductor (CMOS) and charge-coupled devices (CCDs) driven by global market demands, digital cameras and cellphones can take pictures containing millions of pixels using a chip not larger than a fingernail.

Given the fact that the number of pixels in a camera sensor has already passed twenty million, the purchase of further increasing the pixel number seems to be not only beyond necessity, but also a waste of data storage in conventional applications. Alternatively, it is possible to reconstruct an image with just a single-pixel detector [1,2,3,4] by measuring the total intensity of overlap between a scene and a set of masks using a single element detector, and then combining the measurements with knowledge of the masks. As a matter of fact, if one looks back more than a hundred years, when detector arrays hadn’t been developed, one would see that scientists and inventors were already endeavoring to retrieve images using just a single-pixel detector, such as an “electric telescope” using a spiral-perforated disk conceived by Paul Nipkow in 1884 [5] and the “televisor” pioneered by John Logie Baird in 1929 [6]. This imaging technique was referred to as “raster scan” and the mathematical theory of image scanning was developed in 1934 [7]. Though no longer the first choice for visible spectrum imaging after the emergence of detector arrays, raster scan systems are commonly used in applications of non-visible spectrums [8,9,10], where detector arrays of certain wavelengths are either expensive or unavailable.

Over the past two decades, so-called “ghost imaging” has reignited the research interests of single-pixel imaging architectures after its first experimental implementation [1]. Originally designed to measure the entanglement of biphotons emitted from a spontaneous parametric down-conversion (SPDC) light source with two bucket detectors (a combination of a single-pixel detector and a collecting lens) for imaging purposes [11,12], it was soon demonstrated, by thermal light experiments [13,14,15,16] and computational ones [2,4,17], that ghost imaging is not quantum-exclusive but a variant of classical light field cross-correlation [18,19].

However, ghost imaging is sometimes referred to as a quantum-inspired computational imaging technique [20], where “computational” means that the imaging data measured by a ghost imaging system needs to be processed by computational algorithms before it can actually look like a conventional image. While using a single-pixel detector in the imaging hardware may offer a better detection efficiency, a lower noise, and a higher time resolution than a pixelated detector array does, the computational algorithms introduce competitive edges from the software perspective, due to ever-increasing processing power. One such example is compressive sensing [21,22,23,24], which allows the imaging system to sparsely measure the scene with a single-pixel detector and consequently to collect, transfer, and store a smaller amount of image data. This imaging technique using compressive sensing became known as single-pixel imaging [3].

**Figure 1:**
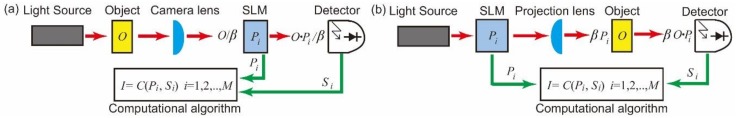
Schematics of two imaging architectures: (**a**) In single-pixel imaging, the object is first illuminated by the light source, then imaged by a camera lens onto the focal plane, where a spatial light modulator (SLM) is placed. The SLM modulates the image with different masks, and the reflected or transmitted light intensities are measured by a single-pixel detector. A computational algorithm uses knowledge of the masks. along with their corresponding measurements, to reconstruct an image of the object; (**b**) in ghost imaging, the object is illuminated by the structured light distribution generated from different masks on an SLM, and the reflected or transmitted light intensities are then measured by a single-pixel detector. A computational algorithm uses knowledge of the masks along, with their corresponding measurements, to reconstruct an image of the object.

It soon became obvious to both the single-pixel imaging community and the ghost imaging community that the two imaging architectures are essentially the same in an optical sense, as shown in Figure 1, where single-pixel imaging (Figure 1a) places a spatial light modulator (SLM) on the focal plane of the camera lens to modulate the image of the scene with different masks before measuring the light intensities with a single-pixel detector, while ghost imaging (Figure 1b) uses different structured light distributions generated by the SLM to illuminate the scene and measures the reflected or transmitted light intensities. The object and the SLM are conjugated by the lens between them in both architectures.

To avoid confusion, both ghost imaging and single-pixel imaging are hereafter referred to as single-pixel imaging. Though not performing as well as detector array in conventional visible imaging, single-pixel imaging has been demonstrated for multi-wavelength imaging [25,26,27,28,29], terahertz imaging [30,31], X-ray imaging [32,33,34], temporal measurement [35,36,37], and three-dimensional (3D) imaging [38,39,40,41,42,43,44,45,46,47,48], all of which pose difficulties for conventional cameras. This review focuses on how single-pixel imaging can be utilized to obtain 3D information of a scene. The focal plane modulation architecture (Figure 1a) is used in the following discussion.

## 2. Three-Dimensional Single-Pixel Imaging

### 2.1. Mathematic Interperation of Single-Pixel Imaging

Mathematically, a greyscale image is a two-dimensional (2D) array, in which the value of each element represents the reflectivity of the scene at the corresponding spatial location. If the 2D array is transformed into one dimension, then the image is *I* = [*i_1_*, *i_2_*, …, *i_N_*]^T^, and obtaining an image is all about determining *N* elements in *I*. The easiest way to achieve that is to measure the value of one element at one time and sequentially measure all *N* values to acquire the image, which is the raster scan imaging approach. However, this single-point scanning approach has an image formation time which is proportional to *N* (i.e., the number of the pixels in the image). A better way is to measure *N* elements simultaneously with a detector array containing *N* pixels, which is exactly what modern digital cameras are doing. Unfortunately, detector arrays are not always available for unconventional spectrums and applications, such as ultraviolet- and time-correlated single photon counting, which is when single-pixel imaging comes into play. In single-pixel imaging, the camera lens forms an image *I* onto the surface of an SLM placed at the focal plane of the lens, the SLM modulates the image *I* with a mask *P_i_*, and the single-pixel detector measures the total intensity of the reflected or transmitted light as the inner product of *P_i_* and *I*,

*s_i_* = *P_i_* × *I*, (1)

where *P_i_* = [*p_i1_*, *p_i2_*, …, *p_iN_*] is a one-dimensional (1D) array transformed from the 2D distribution. After the single-pixel system makes *M* measurements, a linear equation set is formed as

*S* = *P* × *I*, (2)

where *S* = [*s_1_*, *s_2_*, …, *s_M_*]^T^ is a 1D array of *M* measurement values, and *P* = [*P_1_*, *P_2_*, …, *P_M_*]^T^ is an *M* × *N* 2D array known as the measuring matrix. The problem of reconstructing the image of the scene becomes a problem of solving *N* independent unknowns (*i_1_*, *i_2_*, …, *i_N_*) using a set of *M* linear equations. To solve Equation (2) perfectly, there are two necessities: (i) *M* = *N* and (ii) *P* is orthogonal, otherwise Equation (2) is ill-posed.

Providing the two necessities are satisfied, the image can be reconstructed by

*I* = *P*^−1^ × *S*. (3)

**Figure 2:**
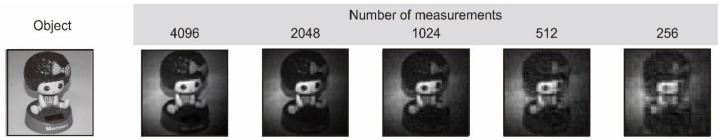
Single-pixel imaging reconstructions (64 × 64 pixel resolution) using experimental data with different numbers of measurements.

The most straightforward choice of the measuring matrix *P* is an identity matrix *E_N_*, which corresponds to the experimental implementation of raster scan imaging systems [3,4,49,50,51]. However, the point-by-point strategy seems inefficient in light of the fact that many natural scenes are sparse or compressible in a way that they can be concisely represented with a proper basis. More importantly, the measuring matrices formed from these sparse bases are orthogonal as well, such as Hadamard [27,52,53,54], Fourier [47,55,56], and wavelet [57,58,59,60], which are commonly used in single-pixel imaging. Consequently, sub-sampling strategies are proposed to sample the scene using *M* smaller than *N*, without jeopardizing the quality of the reconstruction [27,55,57,61]. Figure 2 shows that an image of the scene can be approximately reconstructed with *M* << *N*. It is worth noting that the orthogonal sub-sampling concept, which requires a prior knowledge of the specific scene, is not the same as the compressive sensing, which needs only a general assumption that the scene is sparse. Orthogonal sub-sampling is similar to the idea of image compression techniques such as JPEG [62].

If the measuring matrix *P* is not orthogonal, things become more interesting. In early stages of single-pixel imaging research, the sample masks are (pseudo) random, generated by illuminating a rotating ground glass with a laser beam [10,11], and they form a non-orthogonal measuring matrix. Due to the classical light field cross-correlation interpretation of single-pixel imaging at that time, the reconstruction algorithm simply weights each sampling mask by the magnitude of the corresponding measurement, and then sums these weighted masks to yield the reconstruction of the scene [2,4,10,11,63]. Even using *M* >> *N* during the measuring, the signal-to-noise ratio (SNR) of the yielded images are usually low because of the partial correlation nature of the measurements as well as the lack of sophistication in the reconstruction algorithms. SNR improvement methods are proposed during this stage [64,65,66,67,68,69], among which differential ghost imaging [64] is the most commonly used option.

Fortunately, the pioneering information theory work of Candès and Tao in 2006 demonstrated that by compressively sampling a signal with (pseudo) random measurements, which are incoherent to the sparse basis of the signal, the signal can be recovered from *M* measurements (*M* << *N*) using two approaches: matching pursuit and basis pursuit [70]. This is a perfect match for single-pixel imaging, which uses (pseudo) random masks to sample the scene and requires a large number of measurements to yield a good reconstruction. In a nutshell, for single-pixel imaging via compressive sensing, if the image *I* has an *n*-sparse representation in an *N* orthogonal basis *Q*, and the product of the measuring matrix *P* and the orthogonal basis *Q* (i.e., *P* × *Q*) satisfies the restricted isometry property, then the image *I* can be stably reconstructed from *M* measurements sampled by *P*, where *M* ~ *n*log(*N*/*n*) [71]. The understanding of compressive sensing is not in the scope of this review; those who are interested can refer to the works of Candès, Donoho, and Baraniuk [21,22,23,24].

It is worth mentioning that the time of image formation in single-pixel imaging consists of two parts, acquisition time (i.e., performing *M* measurements) and reconstruction time (i.e., processing the acquired data with a reconstruction algorithm). Compressive sensing reduces the required number of measurements dramatically but has a computational overhead for reconstruction, which limits its application in real-time imaging. Nevertheless, compressive sensing enables the imaging system to perform high dynamic data acquisition [71], provided that the processing of the acquired data is not an immediate requirement. In the case of orthogonal measuring matrices, the reconstruction algorithm usually has a linear iteration nature. Not only is this type of algorithm much less computational compared to those used in compressive sensing, but also the linear iteration can be performed in a multi-thread parallel manner along with the data acquisition, which minimizes the time of image formation. The limitation for the orthogonal measuring matrix strategy is that the required number of measurements increases in proportion to the pixel resolution of the reconstructed image, and it cannot be significantly reduced even if certain adaptive algorithms are utilized [27,57,61].

### 2.2. Performance of Single-Pixel Imaging

If one compares the system architecture of a conventional digital camera to that of single-pixel imaging, one would see that the only difference between them is that the pixelated detector array in a digital camera is replaced by the combination of an SLM and a single-pixel detector. Therefore, the performance of single-pixel imaging is essentially determined by the performance of this combination.

#### 2.2.1. SLM

There are a variety of SLM technologies used in single-pixel imaging, such as a rotating ground glass [10,11], a customized diffuser with pre-designed masks [39,72], a liquid-crystal device (LCD) [2,4], a digital micromirror device (DMD) [27,28], a light-emitting diode (LED) array [48,73], and an optical phased array (OPA) [74,75].

- Spatial resolution

Within the single-pixel imaging approach, the pixel resolution of the reconstructed image is determined by the spatial resolution of the masks, which is limited by the spatial resolution of the SLM used in the system. The spatial resolution of a commonly used DMD module is 1024 × 768, an order smaller than that of a typical commercial digital camera. However, a programmable LCD or DMD offers the flexibility to perform the sampling in various ways, which improves the performance of single-pixel imaging in SNR [53], frequency aliasing suppression [76], or regional resolution [77].

- Data acquisition time

The time to acquire the data of one image in single-pixel imaging is the product of the mask switch time and the number of measurements *M* needed for one reconstruction. DMD, the most common choice in single-pixel imaging, has a typical modulation rate of 22 kHz. Without the help of compressive sensing, it corresponds to a 46.5 ms (1024/22 kHz) acquisition time for 32 × 32 pixel resolution single-pixel imaging, leading to a frame rate of 21 frames-per-second, which is not satisfying. Recent works demonstrated that by using fast-switching photonics components, such as LED array [73] and OPA [74], the modulation rate can be increased beyond 1 MHz, with the potential of reaching GHz.

- Spectrum

For ground glass and customized diffusers, the spectrum they operate in is determined by the materials from which they are made. In the case of LCDs and DMDs, their transmissive or reflective properties decide the bandwidth of the wavelength. In these two circumstances, there is usually a long range of wavelength, which makes wide spectral imaging possible for single-pixel imaging. For LED array and OPAs, the spectrum depends on the light-emitting component, and is usually a narrow-band wavelength.

#### 2.2.2. Single-Pixel Detector

The single-pixel detector is the reason why single-pixel imaging has a much wider range of choices of detection subjects than a digital camera using a detector array does. For starters, single-pixel detectors are available for almost any wavelength throughout the whole electromagnetic spectrum. More importantly, because of the fact that any cutting-edge sensor becomes available in the form of a single-pixel detector long before it can be manufactured into an array, single-pixel imaging systems always enjoy the privilege of using newly developed sensors much earlier than detector array-based conventional cameras do. For example, by using single-pixel detector with single-photon sensitivity, single-pixel imaging systems will be able to image objects much farther away than conventional digital cameras can.

However, these privileges come with a price; that is, with only one detection element, the measurements needed to reconstruct an image must be performed sequentially over a period of time, while they could be performed easily in one shot using a detector array. To compensate for this disadvantage, fast-modulating SLMs, high-speed electrics, and powerful computational capabilities are needed for the single-pixel imaging technique.

**Figure 3:**
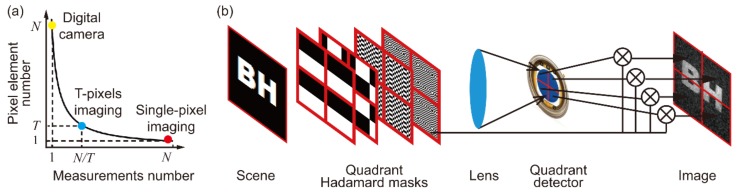
Space–time trade-off relationship for performing *N* measurements: (**a**) Single-pixel imaging and digital cameras are at the two ends of the curve, while the idea of using *T* pixels and *N*/*T* measurements is a compromise between the two extremes; (**b**) by using a quadrant detector, the imaging system is 4 times faster in data acquisition [79].

An interesting idea [78,79] worth mentioning is that one can always make a compromise between two extreme measuring manners; that is, rather than performing *N* measurements using either one pixel with *N* measurements or *N* pixels with a single measurement, the same number of measurements can be achieved by using *T* pixels with *N*/*T* measurements, as shown in Figure 3a. By adopting this idea in single-pixel imaging (Figure 3b), the acquisition time can be reduced by a factor of *T*, though “single-pixel imaging” might no longer be an appropriate name for the imaging system.

Before going any further, a summary is provided in Table 1, in which major elements of a single-pixel imaging system are summarized; their possible choices and corresponding pros and cons are listed.

### 2.3. From 2D to 3D

3D imaging is an intensively explored technique, which is applied to disciplines such as public security, robotics, medical sciences, and defense [80,81,82,83,84,85]. A variety of approaches have been proposed for different applications, among which time-of-flight [50,51,86] and stereo vision [87,88,89,90,91,92] are commonly used. In the following, we review how single-pixel imaging adopts these two approaches to go from 2D to 3D.

#### 2.3.1. Time-of-Flight Approach

Time-of-flight measurement determines the distance *d* to a scene by illuminating it with pulsed light and comparing the detection time *t_a_* of the back-scattered light to the time of the illumination pulse *t*_0_ (i.e., *d* = Δ*tc*/2), where Δ*t* = (*t_a_*−*t*_0_) is the time of flight and *c* is the speed of light. For single-pixel imaging, if the distance information can be obtained at each spatial location of the scene, then a 3D image can be reconstructed by combining a depth map (i.e., the 2D array of distance information) with a transverse reflectivity image of the scene. However, with the flood illumination implemented in single-pixel imaging, the illuminating pulsed laser back-scattered from a scene is significantly broadened, providing only an approximate distance of the whole scene in conventional time-of-flight understanding. Methods for extraction of depth information of each spatial location from a series of broadened pulsed signals are described as follows.

**Figure 4:**
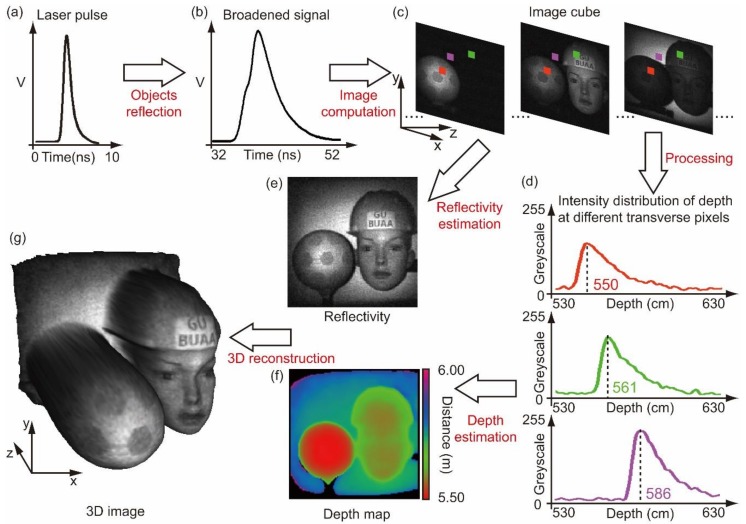
Overview of the image cube method: (**a**) The illuminating laser pulses back-scattered from a scene are measured as (**b**) broadened signals; (**c**) an image cube, containing images at different depths, is obtained using the measured signals; (**d**) each transverse location has an intensity distribution along the longitudinal axis, indicating depth information; (**e**) reflectivity and (**f**) a depth map can be estimated from the image cube, and then be used to reconstruct (**g**), a 3D image of the scene. Experimental data used in this figure is from the work of [45].

In 2D single-pixel imaging, one mask only corresponds to one measured intensity. However, by using pulsed light for illumination and a time-resolving detector for detection, one mask will correspond to a series of measured intensities at different depths. Consequently, a series of images can be obtained by associating the masks with the measured intensities at different depths, forming an image cube in 3D. By further processing the data in the image cube, both reflectivity and depth information of the scene can be extracted, and therefore a 3D image is reconstructed. Many works utilized this concept [38,39,40,41,43,45,72], among which [45] demonstrated its merits most. Figure 4 illustrates the procedure of this method.

This method is straightforward in a physical sense, because the image cube is a 3D array which is also the collection of temporal measurements at each spatial location, that is, the measured data of the raster scan imaging system with a time resolving detector [50,51]. However, the image cube method is computational, because all images at different depths are reconstructed. Therefore, 2D image reconstruction using an orthogonal measuring matrix might be a wise choice, while utilizing compressive sensing would only further burden the data processing.

An alternative method [40] to recover the depth map is abstract in its physical sense but computationally elegant and efficient. Instead of trying to recovering a depth map *I_D_* directly, the method considers a signal *I_Q_* made up of the element-wise product *I_Q_ = I.I_D_*, where *I* is a 2D reflectivity image of the scene obtained by standard single-pixel imaging. More importantly, it is proved that *I_Q_* satisfies the following equation:

*S_Q_* = *P* × *I_Q_*, (4)

where *S_Q_* = [Σ*^J^_j_*_=1_(*s_1,j_ t_j_*), Σ*^J^_j_*_=1_(*s_2,j_ t_j_*), …, Σ*^J^_j_*_=1_(*s_M,j_ t_j_*)]^T^ is a 1D array of the sum of the products between the number of received photons *s_i,j_* at time *t_j_* for the *i*th mask measurement. Therefore, by using the same treatment, a second “image” *I_Q_* can be reconstructed, and dividing by *I*, a depth map *I_D_* of the scene can be yielded as well. Again, it is worth noting that this image *I_Q_*, which is the element-wise product of the reflectivity image *I* and the depth map *I_D_*, does not have a straightforward physical meaning. In this method, only two image reconstructions are performed, and compressive sensing could be utilized without adding too much computational burden.

The performance of the time-of-flight based 3D single-pixel imaging is related to the following aspects. The aspects affecting 2D single-pixel imaging performance are not mentioned here.

- Repetition rate of the pulsed light: One pulse corresponds to one mask measurement, therefore the higher the repetition rate is, the faster an SLM displays the set of masks.
- Pulse width of the pulsed light: A narrower pulse width means a smaller uncertainty in time-of-flight measurement and less overlapping between back-scattered signals from objects of different depths, which in turn improves the system depth resolution.
- The type of the single-pixel detector: The choice of whether to use a conventional photodiode or one operated with a higher reverse bias (e.g., a single-photon counting detector), is dependent on the application. A single-photon counting detector, which can resolve single-photon arrival with a faster response time, is well suited for low-light-level imaging. However, its total detection efficiency is very low since only one photon is detected for each measuring pulse. Furthermore, the inherent dead time of the single-photon counting detector, often 10s of nanoseconds, prohibits the information retrieval of a farther object if a closer one has a relatively higher detection probability. In contrast, a high-speed photodiode can record the temporal response from a single illumination pulse, which can be advantageous in applications with a relatively large illumination.
- Time bin and time jitter of the electronics: These two parameters are usually closely related, and the smaller they are, the better the depth resolution will be. However, a smaller time bin also means a larger amount of data, which will burden the reconstruction of the 3D image.

A major advantage of time-of-flight-based 3D imaging over other 3D imaging techniques, such as stereo vision [87,88] or structured-light 3D imaging [93,94], is that time-of-flight measurement is an absolute measurement, meaning that the depth resolution of time-of-flight-based 3D single-pixel imaging systems are not largely affected by increases in the distance between the system and the object. Therefore, it is a good candidate for long-distance 3D measurement, such as LiDAR [83].

#### 2.3.2. Stereo Vision Approach

Stereo vision uses two or more images obtained simultaneously from different viewpoints to reconstruct a 3D image of the scene. However, the geometry registration between several images during the reconstruction can be problematic. Contrarily, photometric stereo [89,90,91,92] captures images with a fixed viewpoint but different illuminations. The pixel correspondence in photometric stereo is easier to perform than in stereo vision, but images of different illumination have to be taken sequentially, which limits its real-time application. 

**Figure 5:**
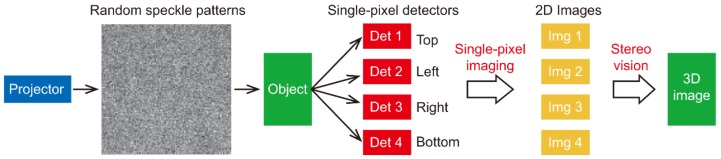
Schematic of a stereo vision based 3D single-pixel imaging.

In 3D single-pixel imaging utilizing stereo vision, as show in Figure 5, a digital projector illuminates the object with random speckle masks. Four single-pixel detectors, placed above, below, and to the left and right sides of the projector, measure the back-scattered light intensities. Four images of the viewpoints are obtained by associating the measured data with knowledge of the random speckle masks. A 3D image of the object is reconstructed from four images and the geometry information of their corresponding viewpoints. The single-pixel imaging fundamentally functions as conventional digital cameras do in the system, however, the architecture of single-pixel imaging enables the simplification of the 3D imaging system to only one SLM, one camera lens, and several pixels without compromising the quality of the reconstruction [42]. More importantly, with a concise system setup, both simultaneous capture of images and pixel correspondence among images can be easily addressed [92]. 

The performance of stereo vision-based 3D single-imaging is essentially determined by two aspects, the quality of the viewpoint’s 2D images and the stereo vision geometry of the system setup. For example, both 3D reconstruction quality and speed are improved in [92] compared to [42]. The improvements are mainly achieved by implementing orthogonal measuring masks with an SLM of a higher modulation rate. A recent work replaces the SLM with an LED array [48], further lowering the system cost. The depth resolution of the system is a relative quantity, which is largely constrained by the geometry of the system setup, in particular, the ratio of the separation between the single-pixel detectors to their distance to the object. Applications such as close industrial inspection and object 3D profiling would be suitable for stereo vision-based 3D single-imaging.

## 3. Conclusions and Discussions

In this review, we briefly go through the development of the single-pixel imaging technique, provide a mathematical interpretation of its working principles, and discuss its performance from our understanding. Two different approaches to 3D single-pixel imaging, and their pros, cons, and potential applications are discussed.

The potential of the single-pixel imaging technique lies in three aspects of its system architecture. First, the use of single-pixel detectors makes single-pixel imaging a perfect playground for testing cutting edge sensors, such as single-photon counting detectors, in imaging techniques. Furthermore, it offers an easy platform from which to adopt other single-pixel based techniques, for example, the spatial resolution of the ultrafast time-stretch imaging [95,96,97,98] could be enhanced by adopting single-pixel imaging in time domain.

Second, the use of programmable SLMs provides extra flexibility for the imaging formation. For example, the pixel geometry of the image can be arranged in non-Cartesian manners, and the trade-off between SNR, spatial resolution, and frame-rate of the imaging system can be tuned according to the demands of the application. SLMs also set limitations on the performance of single-pixel imaging with their own spatial resolutions and modulation rates. Therefore, high performance SLM devices are desirable for the development of the single-pixel imaging technique. 

Third, the use of compressive sensing enables imaging reconstruction using sub-sampling without following the Nyquist’s theorem, resulting in a significant decrease in the amount of data during the image acquisition and transfer, rather than compressing the acquired image after it is sampled completely. Sooner or later, with the ever-growing capabilities of processors, the computational burden of compressive sensing algorithms will not be a limitation.

Despite the fact that the current performance of single-pixel imaging is not comparable, particularly in visible spectrum, to that of conventional digital cameras based on detector array, it is a fascinating research field in which to test cutting edge sensors and experiment with new imaging concepts. Unlike digital cameras, single-pixel imaging doesn’t have any components which are specially developed for it due to global market demands and the funding that follows. However, the situation might be changing with the emerging need for low-cost 3D sensing techniques for autonomous vehicles. 

## Figures and Tables

**Table 1:** Summary of major elements in single-pixel imaging.

Element	Choices	Advantages (*) and Disadvantages (^)
System architecture	Focal plane modulation	* Active or passive imaging. ^ Limited choice on modulation.
	Structured light illumination	* More choices for active illumination. ^ Active imaging only.
Modulation method	Rotating ground glass	* High power endurance; cheap. ^ Not programmable; random modulation only.
	Customized diffuser	* High power endurance; can be customized. ^ Not programmable; complicated manufacturing.
	LCD	* Greyscale modulation; programmable. ^ Slow modulation; low power endurance
	DMD	* Faster than LCD; programmable. ^ Binary modulation; not fast enough.
	LED array	* Much faster than DMD; programmable. ^ Binary modulation; structured illumination only.
	OPA	* Much faster than DMD; controllable. ^ Random modulation; complicated manufacturing.
Reconstruction algorithm	Orthogonal sub-sampling	* Not computationally demanding. ^ Requires a specific prior.
	Compressive sensing	* A computational overhead. ^ Needs only a general sparse assumption.
